# Efficacy of sirolimus for the prevention of recurrent pneumothorax in patients with lymphangioleiomyomatosis: a case series

**DOI:** 10.1186/s13023-018-0915-2

**Published:** 2018-09-21

**Authors:** Li Zhou, Ruoyun Ouyang, Hong Luo, Siying Ren, Ping Chen, Yating Peng, Ting Liu, Guiqian Liu

**Affiliations:** Department of Pulmonary and Critical Care Medicine, The Second Xiangya Hospital, Central South University, 139 Renmin Road, Changsha, China

**Keywords:** Sirolimus, Lymphangioleiomyomatosis, Pneumothorax, Treatment strategy, Recurrence

## Abstract

Pneumothorax is one of the most common symptoms in patients with lymphangioleiomyomatosis (LAM). However, current management strategies for patients with LAM who present with recurrent pneumothorax remain inadequate. Here, we describe the successful prevention of recurrent pneumothorax by sirolimus treatment in five women with LAM. Before sirolimus treatment, all patients had received supplemental oxygen support, repeated chest tube drainage, or surgeries for management of the recurrent pneumothorax. Sirolimus treatment was initiated when the pneumothorax was completely resolved, and no patient developed pneumothorax during treatment. Moreover, they exhibited a significantly improved subjective quality of life, increased exercise capacity, and mild adverse effects such as mucositis, irregular menstruation, and delayed wound healing. On discontinuation of sirolimus or in the event that the plasma sirolimus level was markedly low, pneumothorax tended to relapse. The findings from these cases provide valuable insights that will aid in the improvement of treatment strategies for patients with LAM and recurrent pneumothorax.

## Introduction

Lymphangioleiomyomatosis (LAM) is a rare, progressive, cystic lung disease that mostly affects women of child-bearing age and is characterized by abnormal smooth muscle cell proliferation. LAM is associated with a series of clinical manifestations such as dyspnea, recurrent pneumothorax, hemoptysis, chylous effusion, renal angiomyolipoma (AML), retroperitoneal masses, and respiratory failure [1]. Pneumothorax is a common manifestation of LAM. Previous studies have demonstrated that approximately 66% of patients with LAM may exhibit pneumothorax; importantly, 70% of these patients may experience recurrent ipsilateral or contralateral pneumothoraces [2, 3]. However, management strategies for recurrent spontaneous pneumothorax in patients with LAM remain controversial and inadequate. Sirolimus, a common mammalian target of rapamycin (mTOR) inhibitor, is considered the first effective drug for patients with LAM. According to the guidelines published in 2016 [4], sirolimus is recommended for the following types of patients with LAM: patients with moderately impaired lung function [a forced expiratory volume in 1 s (FEV1) of less than 70% predicted] or progressively declining lung function (decline rate for FEV1, over 90 ml/year) and patients with chylous effusion. However, to date, sirolimus has not been recommended for patients with LAM presenting with pneumothorax. Here, we describe the clinical course of five women with LAM who presented with recurrent pneumothorax that was successfully prevented by sirolimus treatment. We also discuss the effectiveness of sirolimus therapy and other therapeutic options for the prevention of pneumothorax recurrence in patients with LAM, in an attempt to promote the development of better treatment strategies for this patient population.

### Case series

## Case 1

A 33-year-old female nonsmoker with a 4-month history of intermittent chest pain and dyspnea at rest, which recurred every 2 weeks, was admitted to our hospital at 31 weeks of gestation. Four months ago, she had been admitted after experiencing these symptoms for the first time. A chest radiograph at that time revealed left hydropneumothorax with 90% lung compression. The patient received closed chest tube drainage (CTD). However, left pneumothorax recurred during rest or minimal activity in the 20th, 25th, 28th, and 30th weeks of gestation. For every recurrent episode, she was admitted to a local hospital, where she received CTD and was discharged only after radiographic confirmation that the pneumothorax had completely resolved. At the current admission, arterial blood gas analysis indicated type I respiratory failure with a partial pressure of oxygen (PaO_2_) of 51 mmHg. The patient was treated with supplemental oxygen and continuous CTD. Between the 31st and 32nd weeks of gestation, abdominal ultrasound revealed that the umbilical cord was twisted around the neck of the fetus. At 33rd weeks, the patient underwent a cesarean section and successfully delivered a baby with a low birth weight of 1720 g and normal Apgar scores. High-resolution computed tomography (HRCT) revealed small, thin-walled cystic lesions diffused throughout all lung fields. The serum level of vascular endothelial growth factor-D (VEGF-D) was 6608 pg/ml. The patient was diagnosed with LAM, and she began treatment at a dose of 2 mg/day from 28 days after delivery. At 18 months after treatment initiation, the patient’s exercise capacity and quality of life exhibited considerable improvement, and she was able to resume work. She was followed up for 3 years and had not experienced recurrent pneumothorax at the time of writing this report. She could perform all daily activities, including jogging, housekeeping, and routine work. The only sirolimus-associated adverse effect was mucositis, which gradually ameliorated and resolved with time during the course of sirolimus treatment. A follow-up pulmonary function test (PFT) revealed a forced vital capacity (FVC) of 2.20 l (75.3% predicted), FEV1 of 1.85 l (66.3% predicted), and an FEV1/FVC ratio of 84.1% (100% predicted). Moreover, she could cover a distance of 480 m in a 6-min walk test (6MWT). Her baby showed normal growth and remained healthy without breast milk.

### Case 2

A 23-year-old female nonsmoker was admitted to our hospital with 6 days of dyspnea. The chest radiograph revealed bilateral hydropneumothoraces (50% and 80% compression in the left and right lungs, respectively). Two months ago, a large intraperitoneal mass had been detected during prenatal examination, and the patient underwent laparatomy with abdominal mass resection and left nephrectomy. Postoperative pathological examination with hematoxylin and eosin (H&E) staining demonstrated that the renal mass consisted of malformed blood vessels, fusiform smooth muscle bundles, and adipose tissue. Immunohistochemical staining revealed positive expression of human melanoma black 45 (HMB45), smooth muscle actin (SMA), and cluster of differentiation 34 (CD34). She underwent bilateral CTD and when her lungs markedly re-expanded, chest HRCT was performed and revealed multiple, diffuse, round, thin-walled cysts in both lungs. TSC gene mutation was not seen. The patient opted for a conservative management strategy with observation and intermittent supplemental oxygen administration after complete resolution of the pneumothorax. However, 4 months later, the patient developed left followed by right pneumothorax. She was hospitalized for over 30 days, and after complete resolution of the pneumothoraces, she began sirolimus treatment. The plasma sirolimus level was maintained at 4–5 ng/ml in repeated measurements. The patient was followed for > 1 year without pneumothorax recurrence, and she could perform all types of ordinary exercises, including running, mountain climbing, cycling, housekeeping, and other outdoor activities. The only sirolimus-associated adverse effect was a mildly intermittent menstruation disorder. A follow-up PFT revealed an FVC of 2.08 l (61% predicted), an FEV1 of 2.04 l (70% predicted), an FEV1/FVC ratio of 98% (87% predicted), a diffusing capacity for carbon monoxide (DLCO) of 4.70 mmol/kPa/min (75% predicted), and a total lung capacity of 3.13 l (68% predicted). She could cover a distance of 550 m in 6MWT. However, the patient discontinued sirolimus after 1.5 years without seeking advice from her physicians because she was planning to conceive for the second time. Three months later, she presented with left chest pain and an uncomfortable feeling in her chest on movement. She received supplemental oxygen support at home for 5 days, following which a chest radiograph revealed left pneumothorax with 30% lung compression. Over the next 2 months, she experienced two episodes of right pneumothorax. She was hospitalized again and remained unable to work or perform regular activities.

### Case 3

A 31-year-old female nonsmoker presented with chest pain and dyspnea at rest. Chest HRCT revealed right pneumothorax with 90% lung compression and multiple bilateral pulmonary bullae. The patient received CTD and oxygen supplementation, followed by bullectomy in the right upper lung lobe. Postoperative H&E staining of lung tissue revealed small spindle-shaped cells distributed alongside bronchioles, blood vessels, and lymph vessels. Immunohistochemical staining demonstrated positive expression of HMB45, SMA, estrogen receptor (ER; 80%), and progesterone receptor (PR; 80%). Considering her age and the fact that it was her first episode of pneumothorax, sirolimus therapy was not initiated. During the following 6 months, the patient presented with unilateral pneumothorax with 30% lung compression and was unable to resume work, and she expressed concern about the recurrence. Nine months later, she was readmitted with bilateral pneumothoraces (compression of the right and left lungs: 95% and 70%, respectively). She successively received supplemental oxygen support, CTD, and left chemical pleurodesis with 50 ml high-sugar + 5-ml lidocaine infusion and autologous blood for sclerification. Eventually, the chest tube was successfully removed, and the patient opted to begin sirolimus treatment at 2 mg/day. Follow-up plasma sirolimus levels ranged from 6 to 10 ng/ml. At the time of writing this report, the patient had been followed up for 2.5 years without recurrence, had resumed her job, and was able to perform ordinary exercises, including jogging, running, mountain climbing, and badminton. A follow-up PFT revealed an FVC of 2.24 l (72.7% predicted), FEV1 of 2.23 l (73.6% predicted), and FEV1/FVC ratio of 99% (118% predicted), and she could cover a distance of 555 m in 6MWT.

### Case 4

A 38-year-old female nonsmoker with an 8-year history of recurrent dyspnea and hemoptysis, which had been aggravated since a week, was admitted to our hospital. She had experienced right chest pain and mild dyspnea following a sneeze 8 years ago, and chest radiography at that time confirmed right pneumothorax. Chest HRCT revealed bilateral, diffuse, round, thin-walled cysts with varying sizes. She was clinically diagnosed with tuberculosis and received anti-tuberculosis therapy for 6 months. Two years later, the patient underwent pleurodesis under video-assisted thoracoscopic surgery (VATS) because of recurrent pnuemothoraces and for further evaluation of the thin-walled cystic lesions. Postoperative pathological examination of lung tissue revealed the characteristics of pulmonary LAM. Immunohistochemical examination demonstrated positive expression of HMB45, SMA, ER, and PR. Over the next 3 years, she experienced recurrent pneumothorax, mainly in the right lung. At the current admission, the chest radiograph revealed right pneumothorax with 60% lung compression. Two days later, the patient exhibited severe dyspnea with cyanotic lips and nails, and unconsciousness. Arterial blood gas analysis revealed type I respiratory failure with a PaO_2_ of 45.5 mmHg, while a chest radiograph showed massive bilateral pneumothoraces. On resolution of the pneumothoraces, the patient opted to begin sirolimus treatment at 2 mg/day. She was diagnosed with recurrent tuberculosis at the same time, and anti-tuberculosis therapy was also initiated. The plasma sirolimus level was 3.9 ng/ml. Following anti-tuberculosis treatment for 1 year, her respiratory symptoms completely resolved, and the treatment was discontinued. One month later, she presented with swollen, painful ankles and fingers. The plasma sirolimus level at that time was > 15 ng/ml. The findings of rheumatological and immunological examinations were unremarkable. The ankle and finger pain was considered a side effect of sirolimus treatment; therefore, the dose was reduced to 1 mg/day. The ankle pain resolved; however, the patient developed slight fever, and clinical examinations revealed that the tuberculosis had relapsed. Accordingly, anti-tuberculosis therapy was restarted. Four months later, she experienced recurrent dyspnea and right pneumothorax; the plasma sirolimus level was 0.01 ng/ml. The sirolimus dose was increased to 2 mg/day, and she additionally received supplemental oxygen support. Two months later, the plasma sirolimus level was 2.97 ng/ml, and a chest radiograph revealed complete resolution of the pneumothorax. Sirolimus-associated side effects included mild mucositis, joint pain, and menoxenia. At the time of writing this report, the patient had been followed up for > 3 years, with gradually improved respiratory symptoms. She exhibited an improved quality of life and was able to perform daily activities such as housework, jogging, cycling, and mountain climbing. A follow-up PFT revealed an FVC of 3.39 l (120.2% predicted), FEV1 of 2.38 l (90.5% predicted), and FEV1/FVC ratio of 70.2% (86.2% predicted). The distance covered in 6MWT was 510 m.

### Case 5

A 30-year-old female smoker presented with a 3-year history of recurrent pneumothorax, chest pain, and dyspnea on exercise. Chest CT obtained after the first episode of pneumothorax 3 years ago revealed left pneumothorax with 50% lung compression and bilateral, multiple, thin-walled pulmonary cysts. The patient underwent left pulmonary bullectomy and intrapleural fixation. However, the patient still experienced frequent left or right pneumothorax at rest or on minimal activity, although it showed spontaneous resolution. A year ago, the patient was admitted to a local hospital with severe pain in the right chest and dyspnea. A chest radiograph revealed right pneumothorax with 30% lung compression. She also developed recurrent lower abdominal pain accompanied by nausea and vomiting. Abdominal magnetic resonance imaging revealed multiple retroperitoneal cystic masses (15.6 × 20.2 cm), a cystic mass at the right uterine attachment (6.2 × 3.6 × 7.0 cm). She underwent retroperitoneal tumor resection, and postoperative pathological examination of the retroperitoneal mass revealed a large number of spindle-shaped cells distributed alongside blood vessels and lymph vessels. The cells showed no obvious heterotypic features, necrosis, and mitosis. Immunohistochemical examination demonstrated positive expression of SMA, HMB45, ER, PR, and D2–40. The serum VEGF-D level was 2685.88 pg/ml. Considering the possibility of recurrent pneumothorax, the patient agreed to begin sirolimus therapy at 1 mg/day. At the time of writing this report, the patient had been followed up for 5 months without recurrent pneumothorax or abdominal pain. A follow-up PFT revealed an FVC of 3.12 l (93.6% predicted), an FEV1 of 2.35 l (81.4% predicted), an FEV1/FVC ratio of 75.54% (84.06% predicted), a DLCO of 5.35  mmol/kPa/min (61.4% predicted), and a total lung capacity of 4.31 l (93% predicted). The plasma sirolimus levels in the first and third months of treatment were 5.28 and 7.25 ng/ml, respectively. The distance covered in 6MWT was 480 m. Mild mucositis was the only sirolimus-associated adverse effect.

## Discussion

**Fig. 1:**
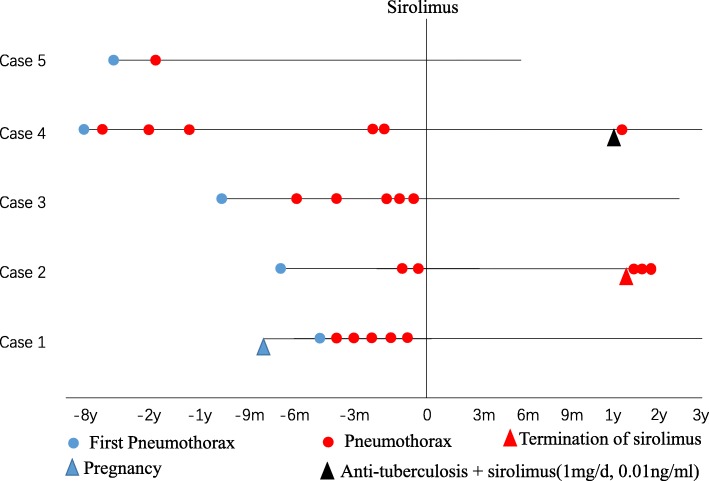
Timelines of pneumothorax recurrence in five patients with lymphangioleiomyomatosis (LAM) who were treated with sirolimus. All five patients had recurrent pneumothoraces before sirolimus treatment. The pneumothorax was induced by pregnancy in case 1. During sirolimus treatment, no patient developed pneumothorax. However, when the patients discontinued sirolimus or exhibited an undetectable trough level due to interaction with anti-tuberculosis drugs, the pneumothorax relapsed

In this report, we described the successful prevention of recurrent pneumothorax by sirolimus treatment in five women with LAM. The clinical characteristics of the five LAM patients with recurrent pnuemothoraces are described in Table 1, and the time course of pneumothorax recurrences is shown in Fig. 1. Spontaneous recurrent ipsilateral or contralateral pneumothorax during rest or minimal activity is one of the most common manifestations, accounting for two-thirds of LAM patients [5]. In a previous study, the majority of patients with LAM initially presented with unilateral pneumothorax, and only 4% initially presented with simultaneous bilateral pneumothoraces [3]. After the first episode of pneumothorax, conditions such as Birt–Hogg–Dubé syndrome, pulmonary Langerhans cell histiocytosis, pulmonary bullae, lymphoid interstitial pneumonia, Sjögren syndrome, and amyloidosis, all of which are characterized by diffuse, thin-walled cystic lesions in the lungs on HRCT, should be ruled out [6]. All five patients reported here developed pneumothorax before LAM was diagnosed, and chest pain and dyspnea were the most frequent symptoms, and two of the patients experienced type I respiratory failure. In addition, all patients experienced recurrent homolateral or contralateral pneumothorax, which resulted in a poor quality of life and repeated hospitalizations. The management of recurrent pneumothorax in patients with LAM has been controversial. The latest official guideline from the American Thoracic Society/Japanese Respiratory Society recommends that ipsilateral pleurodesis should be performed when patients with LAM experience their first episode of pneumothorax (conditional recommendation, very low confidence in the estimated effects) [7]. An observational study of 395 patients registered with the LAM Foundation [3] revealed that two-thirds of patients presenting with pneumothorax subjected to conservative therapy for the first episode experienced recurrent pneumothorax, with recurrence rates of 32% and 27% for patients who underwent surgical management and chemical pleurodesis, respectively, for the first episode. However, approximately 62% of patients with LAM and pneumothorax select oxygen supplementation or CTD for the first episode, while 60% select pleurodesis for the second episode [2]. All of our patients received conservative treatment such as supplemental oxygen and small-bore chest drain insertion for re-expansion of their lungs after the first episode of pneumothorax, with three of them receiving chemical pleurodesis, surgical pleurodesis, and/or bullectomy after frequent recurrences. However, all five patients continued to develop recurrent ipsilateral, contralateral, or bilateral pneumothoraces despite conservative or aggressive surgical treatment. The efficacy of conservative treatment and pleurodesis for the prevention of pneumothorax recurrence in patients with LAM remains unsatisfactory. Repeated pneumothorax negatively influences the life quality of patients with LAM and significantly increases their healthcare burden. Therefore, it is necessary for physicians to identify an effective drug that can prevent the relapse of pneumothorax and improve the quality of life.

**Table 1:** Clinical data for five patients with lymphangioleiomyomatosis (LAM) and recurrent pneumothorax treated with sirolimus

Characteristics	Case 1	Case 2	Case 3	Case 4	Case 5
Age (years)	33	23	31	38	30
VEGF-D (pg/ml)	6608	2385	1139	3901	2685.8
PaO_2_(mmHg)	51	92	96	45.5	98
Surgery	No	No	Yes	Yes	Yes
Chemical pleurodesis	No	No	Yes	No	No
Number of PTX episodes before sirolimus therapy	6	3	6	6	2
Sirolimus concentration (ng/ml)	5–7	4–5	6–10	3–8	5.28^a^
Number of PTX episodes during sirolimus therapy	0	0	0	1	0
Follow-up 6MWT results (m)	480	550	555	510	500

^a^Only examined once

VEGF-D vascular endothelial growth factor-D, *PTX* pneumothorax, *PFT* pulmonary function test, *6MWT* 6-min walk test, *FVC* forced vital capacity, *FEV1* forced expiratory volume in 1 s, *DLCO* diffusing capacity for carbon monoxide

The findings from the five cases reported here suggest that sirolimus is a promising and effective drug for the prevention of recurrent pneumothorax in patients with LAM. Pneumothorax did not reappear in any of our patients as long as the plasma sirolimus level remained at 3–10 ng/ml. In addition, improvements in the subjective quality of life and exercise capacity were observed during sirolimus treatment for all five patients. All patients were able to resume their daily activities and work during sirolimus therapy. However, the pneumothorax recurred when sirolimus therapy was discontinued or the plasma sirolimus level was very low. The side effects of sirolimus experienced by these patients included the common ones such as mucositis, irregular menstruation, and delayed wound healing. On the basis of our experience, we would suggest that physicians use supplemental oxygen and CTD to facilitate gas discharge and re-expansion of the lungs in patients with LAM who present with pneumothorax. If these conservative methods are not effective, chemical pleurodesis or surgical intervention should be used. Alternatively, physicians can consider pleurodesis as the first choice of treatment for lung re-expansion. Once the pneumothorax has completely resolved and surgical wounds have healed, sirolimus therapy can be initiated as soon as possible to prevent relapse. It should be noted that sirolimus cannot enhance the absorption of pneumothorax and cannot be used to achieve the remission of existing pneumothorax. The potential benefits of sirolimus treatment for patients with LAM and recurrent pneumothorax include an improvement in lung function and the quality of life, an increase in the exercise capacity, and a decrease in the healthcare burden. On conducting a search of PubMed, we found only one case report where pneumothorax in a patient with LAM was successfully treated with sirolimus [8].

We observed that the plasma sirolimus level was remarkably low (0.01 ng/ml) during coadministration of sirolimus at 1 mg/day and anti-tuberculosis therapy in case 4, whereas it ranged between 1 and 3 ng/ml when the patient was receiving sirolimus at 2 mg/day and concomitant anti-tuberculosis therapy. When the patient discontinued anti-tuberculosis therapy, the level of sirolimus was > 15 ng/ml, and obvious adverse effects occurred in the form of pain and swelling of the ankles and fingers. We found that previous findings have suggested a potential interaction between anti-tuberculosis drugs and sirolimus [9, 10]. Therefore, when physicians coadminister sirolimus and anti-tuberculosis drugs, they should individually increase the dose of sirolimus under close monitoring of the plasma levels. In our case series, no patient received talc pleurodesis, considering that previous studies have demonstrated that exposure to talc may increase the risk of lung cancer [11, 12]. Therefore, autologous blood or hypertonic glucose is used as a sclerosing agent for chemical pleurodesis in many hospitals in China because of the low medical risk associated with these agents.

The present case series has some limitations. Because all patients presented to us with pneumothorax, PFT findings prior to sirolimus therapy were unavailable. Therefore, we could not evaluate the efficacy of sirolimus for improving pulmonary function in these patients. Second, pregnancy may have played a role in pneumothorax development in case 1. Therefore, the possibility that pneumothorax may have stopped recurring without sirolimus treatment after pregnancy cannot be completely excluded. Third, we cannot negate the effectiveness of surgical pleurodesis in minimizing recurrences on the basis of our case series. In future, we aim to determine whether sirolimus therapy is more effective than surgery, which is an invasive treatment. Finally, we cannot eliminate bias caused by individual differences in effect of sirolimus.

Thus far, no controlled clinical studies have been conducted for investigation of the possible efficacy of sirolimus for the prevention of pneumothorax recurrence in patients with LAM. In our case series, all patients received supplemental oxygen support, repeated CTD, or surgical treatment for lung re-expansion under case of recurrent pneumothorax before sirolimus treatment, although these strategies proved dissatisfactory in preventing the relapse of pneumothorax. During treatment with sirolimus, however, no patient developed pneumothorax. In addition, they exhibited a significantly improved quality of life. The findings from our case series suggest a potential therapeutic strategy for the management of recurrent pneumothorax in patients with LAM. However, further studies are necessary to clarify our findings.

## Abbreviations

6MWT: 6-min walk test
AML: Angiomyolipoma
AUC: Area under the curve
Cmax: Maximum concentration
CTD: Chest tube drainage
CYP3A4: Cytochrome P450 3A4 isoenzyme
EMA: Epithelial membrane antigen
FEV1: Forced expiratory volume in 1 s
FVC: Forced vital capacity
H&E: Hematoxylin and eosin
HMB45: Human melanoma black 45
HRCT: High-resolution computed tomography
LAM: Lymphangioleiomyomatosis
m-TOR: Mammalian target of rapamycin
PFT: Pulmonary function test
SpO_2_: Blood oxygen saturation
TLC: Total lung capacity
TSC: Tuberous sclerosis complex
VATS: Video-assisted thoracoscopic surgery
VEGF-D: Vascular endothelial growth factor-D
SMA: Smooth muscle actin.
ER: Estrogen receptor
PR: Progesterone receptor
